# Anatomic Interactive Atlas of the Loggerhead Sea Turtle (*Caretta caretta*) Head

**DOI:** 10.3390/ani11010198

**Published:** 2021-01-15

**Authors:** Alberto Arencibia, Aday Melián, Jorge Orós

**Affiliations:** 1 Departament of Morphology, Veterinary Faculty, University of Las Palmas de Gran Canaria, Trasmontaña, Arucas, 35416 Las Palmas, Spain; jorge.oros@ulpgc.es; 2 Daydream Software, Telde, 35200 Las Palmas, Spain; adaymc@gmail.com

**Keywords:** interactive atlas, osteology, dissections, computed tomography, head, anatomy, loggerhead sea turtle, *Caretta caretta*

## Abstract

**Simple Summary:**

Because several diseases have been reported affecting the head of sea turtles, accurate anatomic knowledge of this body part is necessary. We provide an open access, anatomic, interactive atlas of the head of the loggerhead sea turtle (*Caretta caretta*), to facilitate anatomic learning using osteology, gross dissection, and computed tomography (CT) images. Using segmentation and visualization software, relevant anatomic structures were identified and colored in all images, and a computer atlas was developed. This atlas, composed of 55 images, provides an interactive anatomic resource for veterinarians, biologists, researchers, and students involved in loggerhead sea turtle conservation.

**Abstract:**

The head of the sea turtle is susceptible to congenital, developmental, traumatic, and infectious disorders. An accurate interpretation and thorough understanding of the anatomy of this region could be useful for veterinary practice on sea turtles. The purpose of this study was to develop an interactive two-dimensional (2D) atlas viewing software of the head of the loggerhead sea turtle (*Caretta caretta*) using images obtained via osteology, gross dissections, and computed tomography (CT). The atlas is composed of 10 osteology, 13 gross dissection, 10 sagittal multiplanar reconstructed CT (bone and soft tissue kernels), and 22 transverse CT (bone and soft tissue windows) images. All images were segmented and colored using ITK-SNAP software. The visualization and image assessment were performed using the Unity 3D platform to facilitate the development of interactive content in 2D. This atlas can be useful as an interactive anatomic resource for assessment of the head of loggerhead sea turtles.

## 1. Introduction

The loggerhead turtle (*Caretta caretta*) is the most common sea turtle species in the Canary Islands, mainly coming from the US western Atlantic by the Gulf Stream [1]. Currently, the loggerhead turtle is considered as Vulnerable under IUCN Red List criteria, showing a decreasing trend in population globally [2]. Anatomic, physiologic, clinical, and pathologic studies on stranded sea turtles are essential activities for sea turtle conservation around the world [3,4,5,6,7]. Furthermore, in recent decades, the number of veterinary surgeons involved in sea turtle conservation in wildlife rehabilitation hospitals has increased notably [6]. The recent publication of comprehensive books on medicine and surgery in sea turtles and reptiles has been an important help to veterinarians, veterinary students, and veterinary technicians who work with sea turtles [8,9,10,11], but continuing education is also necessary. In addition, the incorporation of “conservation medicine”, a discipline that links animal health with ecosystem health and global environmental change, and “zoological and wildlife medicine” into current and future veterinary curricula at the undergraduate and postgraduate levels has also been supported [12,13].

Many methods have been used to improve the quality of teaching and learning of veterinary anatomy. Resources such as the use of live animals, cadavers, gross dissections, anatomic sections, and plastination enhance anatomic learning by researchers, students, and technicians [14,15,16,17,18]. In recent years, technological developments in the area of computer-assisted learning have improved anatomy teaching [19,20].

ITK-SNAP (Insight toolkit snake automatic partitioning) is a software application used for manual or semi-automatic segmentation of anatomic structures using active contour methods, as well as manual delineation and image navigation. Its primary use is for delineating anatomic structures and regions in computed tomography, magnetic resonance imaging, and other 3D biomedical imaging data facilitating their knowledge [21].

Several different applications of the ITK-SNAP interactive image visualization and segmentation tool have been described. Currently, the use of these new technologies and their associated methodologies has proved to be valuable in the study of human anatomy [22,23,24]. In veterinary medicine, atlas-based segmentation of the canine pelvic limb [25], and canine and ovine brain [26,27] have been described, but no anatomic studies of the loggerhead sea turtle using ITK-SNAP segmentation have been reported.

The head of the sea turtle is susceptible to congenital, developmental, traumatic, and infectious disorders [28,29,30,31,32,33]. An accurate interpretation and thorough understanding of the anatomy of this region could be useful for veterinary practice on sea turtles. Therefore, the objective of this research was to perform an anatomic interactive atlas-based ITK-SNAP segmentation of the head of the loggerhead sea turtle (*Caretta caretta*) using images obtained via osteology, gross dissections, and computed tomography (CT) that may be used as anatomic references for this sea turtle species.

## 2. Materials and Methods

### 2.1. Animals

Six cadavers of juvenile/subadult female loggerhead sea turtles (*Caretta caretta*) that had been stranded in the Canary Islands (Spain) and subsequently died during hospitalization were used for this study. The turtles had been hospitalized at the Tafira Wildlife Rehabilitation Center (TWRC) (Las Palmas de Gran Canaria, Spain) due to severe lesions in rear and/or front flippers. Physical evaluation, including assessments of swimming activity, core body temperature, food ingestion, body weight, straight carapace length (SCL), and hydration, had been performed daily in accordance with a complete clinical assessment protocol. Sea turtle rehabilitation at the TWRC was conducted with the authorization of the Wildlife Department of the Canary Islands Government, in compliance with guidelines of the Ethical Committee for Animal Experimentation (CEEA-ULPGC) (Code: OEBA-ULPGC-02/2016).

### 2.2. CT Technique

A 16-slice Multidetector-row CT (MDCT) scanner (Toshiba Aquilion, Toshiba Medical System, Madrid, Spain) was employed to obtain the CT images of the turtles, which had been placed in ventral recumbence. Transverse CT images were acquired with the following technical parameters: kV 120; mAs 80; collimation and detector configuration 16 × 5, slice thickness 5 mm; recon increment 5 mm; matrix 512 × 512; helical pitch 2; tube rotation time 0.75 s. These images were transferred to a DICOM workstation. We applied bone and soft tissue window settings (WW 2700/WL 350 and WW 120/WL 40, respectively) using a standard DICOM computer software (OsiriX MD, Geneva, Switzerland). In addition, 5 mm thick sagittal multiplanar reconstructions (MPR) were performed from the transverse acquired dataset using a convolution bone kernel (FC30) and a soft tissue kernel (FC64).

### 2.3. Anatomic Evaluation

After imaging, gross dissections and osseous anatomic preparations of the head were used to facilitate accurate anatomic interpretation of the CT images. Clinically relevant anatomic structures were identified and labelled according to internationally accepted veterinary anatomic nomenclature [14,34,35]. Moreover, anatomic structures were photographed, and then were processed for digitalization using a computer program (Adobe^®^ Photoshop^®^ CS5) to improve the quality of the images.

### 2.4. Atlas Technical Methods

#### 2.4.1. ITK-SNAP

Segmentation of the head images was performed using the ITK-SNAP software application [21]. It was used to import osteology, gross dissections, and CT images, and manually delineate the different areas of interest over each image. After this manual process, we created a package for each image. Each package was composed of:

- Full HD image.
- Segmentation file (with the defined anatomic area).
- Information file (containing the color and label of each anatomic structure).

**Figure 1:**
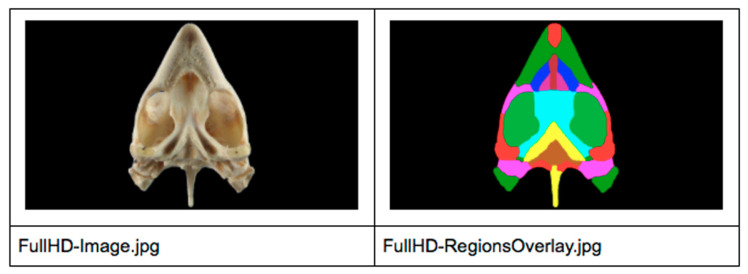
Representative image segmented using the ITK-SNAP software application is shown.

A representative image showing the segmentation using ITK-SNAP software is presented in Figure 1.

#### 2.4.2. 2D Image Digital Processing

Starting from previously created packages, we needed to adapt that information structure so it could be used in an interactive atlas. We had to manually process all the packages of the corresponding 55 images of the atlas, applying the following steps for each of the images:

- Open the package into ITK-SNAP (Full HD image + Segmentation file + Information File).
- Check all region colors were unique so they could be identified later by their colors.
- Check all labels had no typographical errors.
- Define a common image resolution (we used Full HD image max resolution).
- Export Full HD Image as JPG.
- Enable all region colors (unique) and overlay them with full opacity.
- Export Full HD Regions Overlay (same resolution as Full HD image).
- Export Information File (for regions identification).

All packages were located in a folder divided into subfolders, with the image type name and numbered.

Folder (Figure 1):

/Osteology/5/

Full HD–Image.jpg

Full HD–Regions Overlay.jpg

Information File.txt

#### 2.4.3. Unity 3D

Unity is a cross-platform game engine developed by Unity Technologies, which has been extended to support more than 25 platforms. This engine can be used to create three-dimensional, two-dimensional, virtual reality, and augmented reality games, as well as simulations and other experiences. The engine has been adopted by industries outside of video gaming, such as film, automotive, architecture, engineering, and construction [36,37,38].

We decided to use this engine for this project because it has multiresolution support, it supports export as a WebGL application (it can be accessed from web browsers), it can be used to script automatic image treatment for bulk atlas package processing, and we have several years of experience using this software. We divided the project into three big features to create an atlas from the packages, dividing them into their image type name.

#### 2.4.4. Atlas Builder

In order to integrate all ITK-SNAP packages into Unity 3D, we needed to create some C# Scripts into Unity 3D for bulk image processing. We processed all the images at the same time, adapting them to the Unity 3D workflow.

The created script followed this algorithm:

- Select a parent folder with all image types for the atlas.
- Iterate over all image type folders and subfolders.
- Create needed data structures inside Unity 3D for each image found on iteration.
- Clean Full HD images by removing all black pixels from the main image and setting them to transparent.
- Generate Masks: new images for each region with the same resolution reading Information File to obtain the color code, select image pixels using Regions Overlay, and create a mask image with those pixels in white and all others transparent.

Associate these new mask images into Unity 3D data structures with label names.

After applying this script, we had a Unity 3D data structure that we could use to render and create user interaction through all sections.

#### 2.4.5. Atlas Renderer

**Figure 2:**
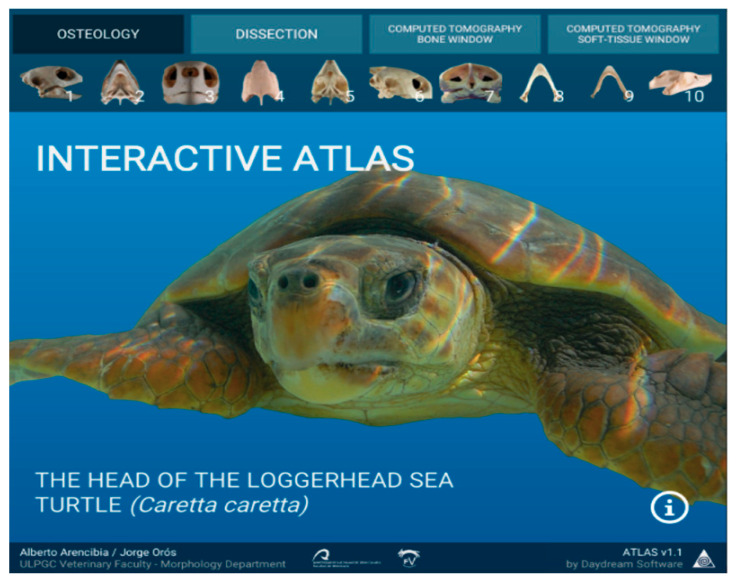
The menu navigation bar is shown for all sections of the atlas.

With all previous generated data, we designed a user interface for easy interaction and navigation through all atlas images. We created a splash image with a top menu navigation for all the image types, which shows below all the available images of that section (Figure 2).

**Figure 3:**
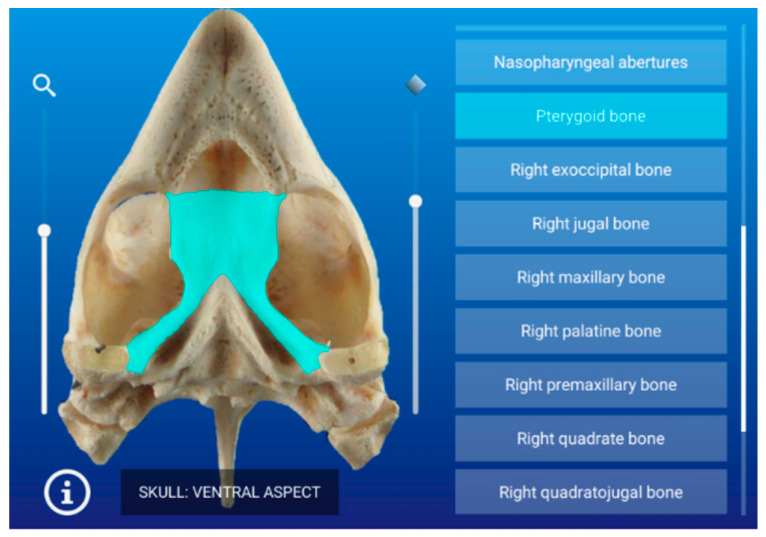
The menu navigation bar is shown for all the labels of a bone image.

After selecting the image, the user enters into the Viewer Area, where all the adapted data are loaded for that image showing all the labels on the right side and the Full HD image on the left side (Figure 3).

We created a double interaction function so if the user selects over the label, the mask image is shown over the left image and if the user moves the mouse over that region, the scroll will look up that mask label and highlight it.

**Figure 4:**
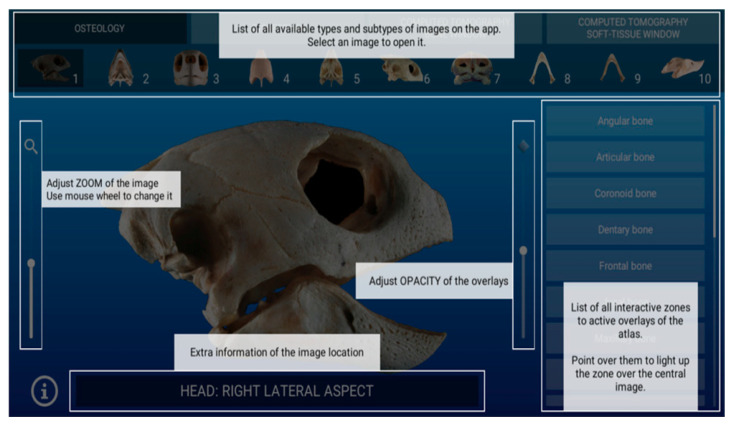
The extra functions of the image viewer are shown.

Additionally, we created some additional functions for easy use on the image viewer such as zoom, masks opacity, and an overlay help panel (Figure 4).

#### 2.4.6. Deployment

We needed to export it for WebGL, therefore we had to optimize the project as much as possible due to limits on current web browsers. We divided the project into external packages from the main app to make it possible to load all images only by demand. This made the project extremely fast to load (compared to previous executions) at the cost of slowing down the image loading time after clicking on the button.

After configuring the server, tweaking some java scripts for use on mobile phones, and editing the HTML page where the WebGL application was loaded, the atlas was ready to use and accessible from all online browsers.

The images of this atlas are available at the following open-access website: http://atlasheadloggerhead.ulpgc.es.

## 3. Results

**Figure 5:**
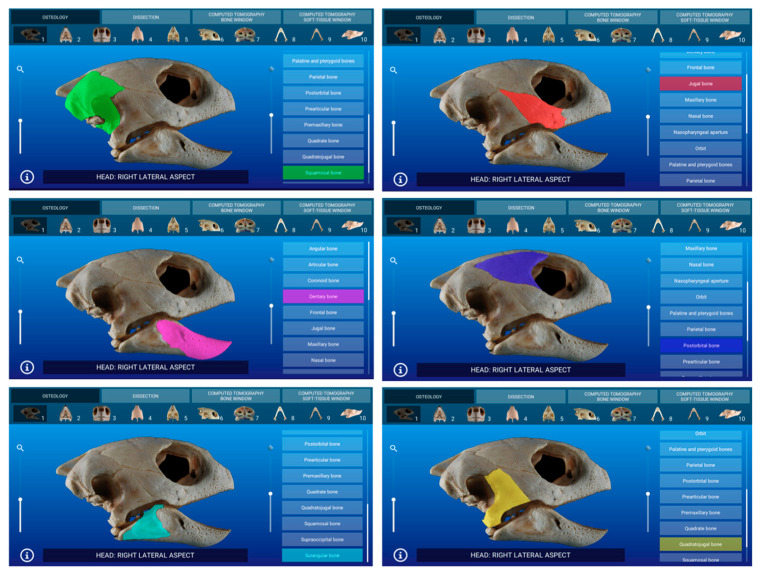
Atlas osteology section: an interactive image of the skull is shown in right lateral aspect.

**Figure 6:**
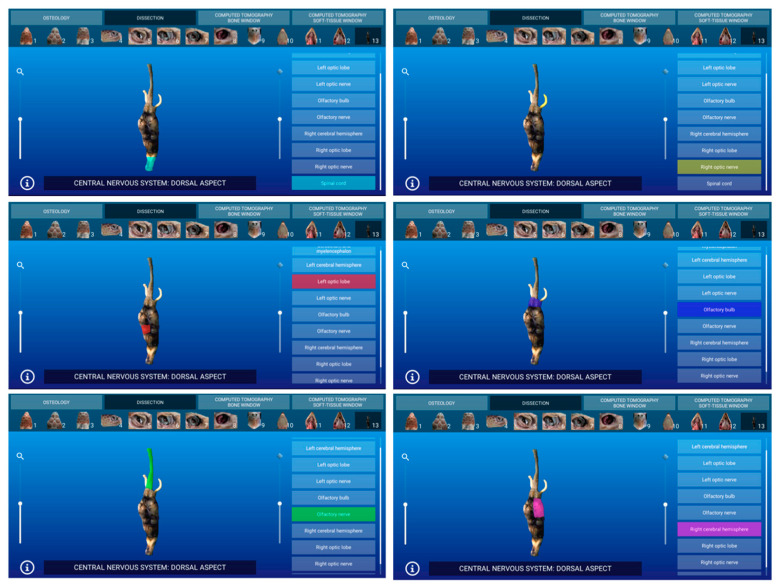
Atlas dissections section: an interactive image of the central nervous system is shown in dorsal aspect.

**Figure 7:**
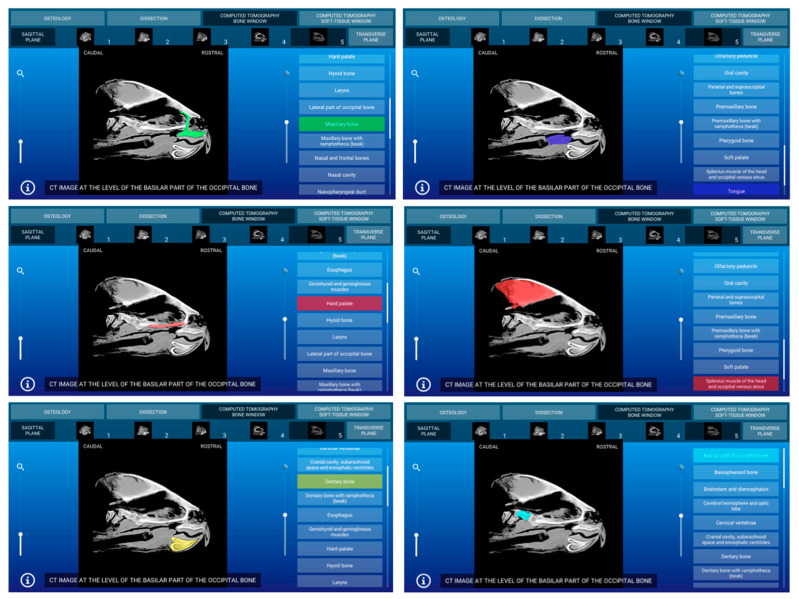
Atlas CT section: an interactive sagittal multiplanar reconstruction (MPR) bone kernel CT image at the level of the prearticular and articular bones is shown in right lateral aspect.

**Figure 8:**
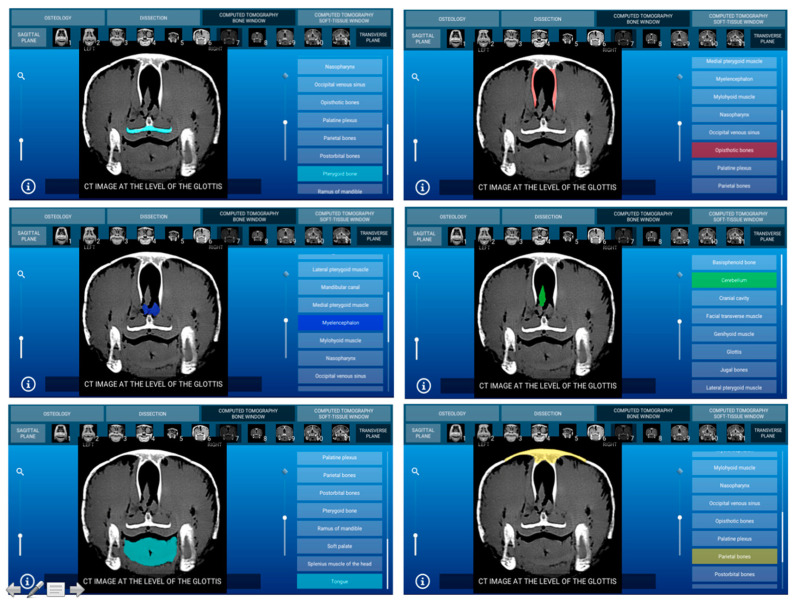
Atlas CT section: an interactive CT bone window transverse image at the level of the glottis is shown in caudal aspect.

**Figure 9:**
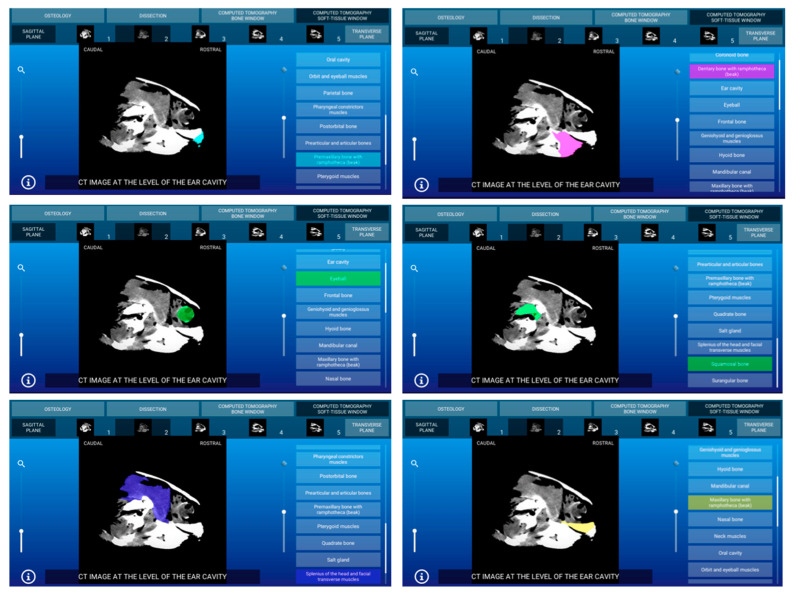
Atlas CT section: an interactive sagittal MPR soft tissue kernel CT image at the level of the ear cavity is shown in right lateral aspect.

**Figure 10:**
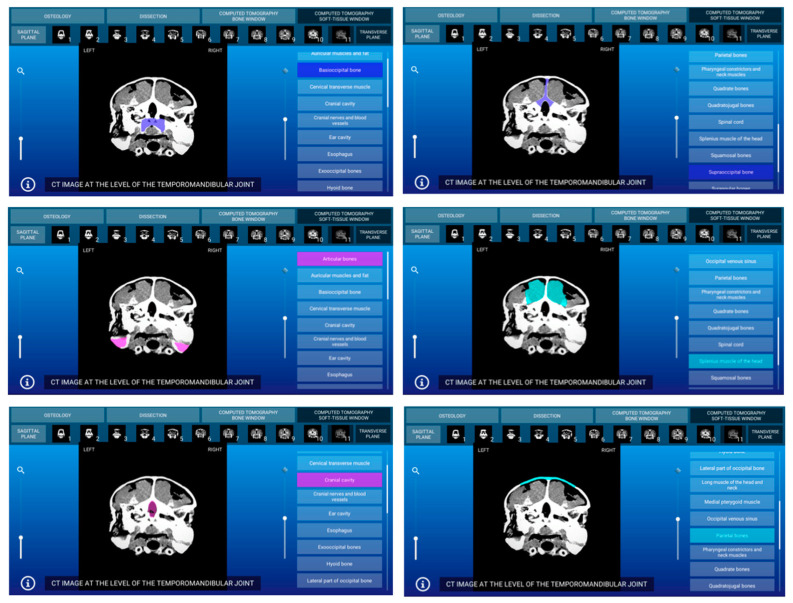
Atlas CT section: an interactive soft tissue window transverse CT image at the level of the temporomandibular joint is shown in caudal aspect.

Representative images of the interactive atlas corresponding to osteology, dissections, and computed tomography sections are presented in Figure 5, Figure 6, Figure 7, Figure 8, Figure 9 and Figure 10. In all images, the main anatomic structures were identified and segmented with different colors.

### 3.1. Osteology

The osteology section of this atlas is composed of 10 images observed in different aspects. The bones of the skull (prefrontal, frontal, parietal, postorbital, supraoccipital, squamosal, quadratojugal, jugal, and maxilla) and mandible (dentary, angular, surangular, prearticular, splenial, and articular bones) were identified in the bone images.

In Figure 5, a representative interactive image of the atlas corresponding to the osteology section shown in lateral aspect is presented.

### 3.2. Dissections

The dissections section of this software is composed of 13 interactive images. The major soft tissues of the head were identified. Thus, bones and head muscles, and most parts of the respiratory (nasal cavity, glottis, and trachea), digestive (oral cavity, tongue, and esophagus), and sensory (eyeball and ear) systems were observed. Additionally, the excretory salt glands were identified. The main components of the brain (telencephalon, diencephalon, mesencephalon, metencephalon, and myelencephalon) were also identified. Other structures such as the rhamphotheca and the scales of the head were also observed.

In Figure 6, a representative image of the atlas corresponding to the dissections section presented in dorsal aspect is observed.

### 3.3. Computed Tomography Images

The CT section of this atlas is composed of two subsections corresponding to the bone window (5 sagittal MPR bone kernel and 11 transverse CT images) and the soft tissue window (5 sagittal MPR soft tissue kernel and 11 transverse CT images) settings. In both CT window settings, the sagittal MPR (bone and soft tissue kernels) CT images are presented in a lateral to medial progression, from the prearticular and articular bones to the basilar part of the occipital bone. These images are presented with the dorsal aspect of the head at the top of the photograph and the rostral aspect of the head on the right side of the photograph. Transverse images are presented in a rostral to caudal progression, from the premaxillary and dentary bones to the temporomandibular joint. These CT images are presented with the dorsal aspect of the head at the top of the photograph and the right side of the head on the right side of the photograph. In the CT images, anatomic details of the head were evaluated according to location and the characteristics of the degree of attenuation of the different tissues with the corresponding bone and soft tissue CT window settings.

#### 3.3.1. CT Bone Window

When applying the bone window setting, the obtained footage showed the best evaluation of the cortical and bone marrow of the bones. Articular sutures and rhamphotheca were clearly observed and appeared with an intermediate degree of attenuation. Air-filled structures of the respiratory (nasal cavity, glottis, and trachea) and digestive (oral cavity and esophagus) appeared with a low degree of attenuation. By contrast, several masticatory, facial, and lingual muscles; excretory salt glands; eyes; and associated structures gave an intermediate CT density and appeared grey. The main nervous structures (myelencephalon, cerebellum, optic lobes, olfactory bulb, and nerves) were clearly appreciated in this modality CT window. Two representative bone window CT images of the atlas corresponding to sagittal MPR bone kernel (Figure 7) and transverse planes (Figure 8) are presented.

#### 3.3.2. CT Soft Tissue Window

With the soft tissue CT window setting, the osseous structures were shown with high attenuation, and differentiation of the cortical bone from the bone marrow was not possible. Articular sutures and rhamphotheca were not clearly observed and appeared with a high degree of attenuation. Air-filled structures of the respiratory (nasal cavity, glottis, and trachea) and digestive (oral cavity and esophagus) systems gave negligible CT tissue density and appeared black. Muscles, excretory salt glands, eyes as well as the main nervous structures gave an intermediate degree of attenuation and appeared grey. Two representative soft tissue window CT images of this atlas corresponding to sagittal MPR (Figure 9) and transverse planes (Figure 10) are presented.

## 4. Discussion

In this study, we developed a digital atlas, as an open access website, of the head of the loggerhead sea turtle. The model was generated using images obtained via osteology, gross dissections, and computed tomography. To the best of our knowledge, this is the first digital interactive atlas of the head of a sea turtle species.

The head of sea turtles is particularly interesting because of the location of the central nervous system (CNS) and important organs such as the eyes, excretory salt glands, ears, mouth, esophagus, nasal cavity, glottis, and trachea [4,14]. It conforms a quite complex anatomic area, which hinders the task of performing physical and clinical assessments of the structures of this region. Several diseases involving the head of sea turtles have been reported. Bone fractures complicated by brain exposure, meningeal hemorrhages, and heterophilic meningoencephalitis due to traumatic lesions mainly caused by boat strikes have been reported [28,31,33,39]; unassisted mortality rate has been recently used as a quality indicator parameter in the rehabilitation of loggerhead turtles, and the highest value was found in the trauma (boat strikes) category, suggesting a poor prognosis for these turtles [6]. Meningitis and encephalitis caused by trematode eggs and adult trematodes have also been described [40,41]. Fibropapillomatosis, characterized by multiple cutaneous fibroepithelial tumors, can affect the head, eye, and esophagus [42,43,44]. Salt gland adenitis as the only cause of stranding has been reported to affect loggerhead turtles [29]. Oral and esophageal lesions caused by hooks [28] and crude oil ingestion [45] have also been reported. According to recent studies on congenital malformations in sea turtles, the head region showed a higher number of malformation types than other body regions [30]. Therefore, precise knowledge of the anatomy of the sea turtle head is necessary to also contribute to the establishment of correct diagnoses. In order to obtain clinical images of the head, CT and magnetic resonance imaging have progressively gained credit for their ability to provide more data to assess the osseous and soft tissue structures of this region [3,4,7,33,46,47]. Diagnostics of several diseases involving the head of loggerhead sea turtles can be improved using CT imaging [7,33,47].

In our study, the CT technique has proven to reliably provide images with good anatomic definition and high contrast among different tissues for this species. For this atlas, a wide window for the bone and a narrow window for the soft tissues were used to obtain the transverse CT images. Both CT windows have provided adequate contrast resolution for bone and for soft tissue, respectively. The CT images allowed us to see the relationship between the medulla and the cortex thanks to the particular bone window settings that we used. In the case of the main soft tissue structures, they could be properly differentiated thanks to the soft tissue window. In addition, MPR sagittal images were obtained using bone and soft tissue kernels. The planimetric CT anatomy of the head allows a correct morphologic and clinical assessment of its anatomic structures in the loggerhead sea turtle [3,7,33,46,47]. The sagittal MPR kernel plane provided the best views of the midline structures of the head, whereas the transverse plane allowed us to better observe the anatomic relationships of the anatomic structures. CT imaging techniques are increasingly available for use in sea turtle veterinary medicine, although the obtainment of images is severely hindered by their hefty cost and limited availability. In addition, the low risk associated with its application could justify its use in these threatened species. The identification of the main structures of the head of the loggerhead turtle in the CT images presented in this atlas was facilitated by the use of bones and the conduction of gross anatomical dissections.

The atlas presents interactive and annotated 2D models of the head, showing 55 individually segmented images using the ITK-SNAP software, which provides semi-automatic segmentation of the anatomic structures using active contour methods, as well as manual delineation and image navigation [21]. Additionally, the interactive ITK-SNAP application reduced analysis time and improved precision in defining anatomic structures as has been reported in other studies [21,22,23,24,25,26,27]. This resource also offers the advantage of color block overlays, providing a clear delineation of structure margins, and digital assignment of labels to an image, thus obviating the need for arrows and complicated label legends [25]. Furthermore, in our study, the visualization and image analysis were performed using the Unity Technologies Real Time 3D Development platform [36,37,38]. In addition, the use of the different tools of this open source software allowed the construction and interactive animation of this atlas.

In our study, the engine Unity 3D was chosen because it provides amazing features for creating any kind of application and publishing it on major platforms [36,37,38]. In this development, we focused on creating a product based on the power of interaction over scientific images. The main core of this product was defined by three key points: ease of access from any device, use of real scientific data and images, and a friendly user experience. Further research on using some JavaScript and HTML5 native solutions instead of Unity 3D, allowing performance of the same functionality and having better performance on any device, is currently being carried out by our group.

Sea turtle conservation has been taken up as a goal by several scientific and academic disciplines, including veterinary medicine [3,4,5,6,7,8,9,10,11,12,13]. The 2D model presented in this research provides a novel, accessible, valuable, intuitive, and interactive anatomic resource for studying the head of the loggerhead sea turtle. It can be a useful tool for specialized veterinarians, biologists, researchers, and technicians involved in sea turtle conservation in wildlife rehabilitation centers around the world, assisting in the interpretation of head diseases in this species.

Finally, this atlas can also be a particularly useful educational tool for academic disciplines incorporated into the veterinary curricula in recent years, such as “zoological and wildlife medicine”, enabling self-learning in an appealing way. Further studies in this sea turtle species are in progress to develop other interactive atlases including different anatomic regions such as the celomic cavity, spinal column, and extremities.

## 5. Conclusions

An interactive atlas of the head of the loggerhead sea turtle has been developed using images obtained via osteology, gross dissections, and computed tomography. The use of this open access interactive atlas could serve as a valid tool for teaching, learning, and training of the anatomy of the head of the loggerhead sea turtle.

## Data Availability

The data presented in this study are available at: http://atlasheadloggerhead.ulpgc.es.
